# Multi-View Laser Point Cloud Global Registration for a Single Object

**DOI:** 10.3390/s18113729

**Published:** 2018-11-01

**Authors:** Shuai Wang, Hua-Yan Sun, Hui-Chao Guo, Lin Du, Tian-Jian Liu

**Affiliations:** 1 School of Graduate, Space Engineering University, Beijing 101416, China; 2 Space Engineering University, Beijing 101416, China; shy221528@vip.sina.com (H.-Y.S.); guohuichaoo@163.com (H.-C.G.); 3 91550 of PLA, Dalian 116000, China; daiqisundu@163.com; 4 63981 of PLA, Wuhan 430311, China; awen871288@163.com

**Keywords:** 3D reconstruction, global registration, loop-closure detection, low-rank sparse decomposition

## Abstract

Global registration is an important step in the three-dimensional reconstruction of multi-view laser point clouds for moving objects, but the severe noise, density variation, and overlap ratio between multi-view laser point clouds present significant challenges to global registration. In this paper, a multi-view laser point cloud global registration method based on low-rank sparse decomposition is proposed. Firstly, the spatial distribution features of point clouds were extracted by spatial rasterization to realize loop-closure detection, and the corresponding weight matrix was established according to the similarities of spatial distribution features. The accuracy of adjacent registration transformation was evaluated, and the robustness of low-rank sparse matrix decomposition was enhanced. Then, the objective function that satisfies the global optimization condition was constructed, which prevented the solution space compression generated by the column-orthogonal hypothesis of the matrix. The objective function was solved by the Augmented Lagrange method, and the iterative termination condition was designed according to the prior conditions of single-object global registration. The simulation analysis shows that the proposed method was robust with a wide range of parameters, and the accuracy of loop-closure detection was over 90%. When the pairwise registration error was below 0.1 rad, the proposed method performed better than the three compared methods, and the global registration accuracy was better than 0.05 rad. Finally, the global registration results of real point cloud experiments further proved the validity and stability of the proposed method.

## 1. Introduction

Flash laser three-dimensional imaging technology [1,2] can obtain an object’s three-dimensional information by transmitting a single pulse, which is an effective means to realize three-dimensional imaging of moving objects. With the advantages of a long imaging distance and freedom from illumination effects, it has high military and civilian value. For example, in the application of space-based non-cooperative object three-dimensional imaging, the object and the imaging system are always in relative motion, and the positions of the two can change at any time. Due to the occlusion of perspective, reconstructing the complete 3D model of the object requires collecting a variety of different perspective point clouds in combination with the object’s motion, and unifying them to the same coordinate system through point cloud registration. Multi-view point cloud registration can be divided into two steps: Pairwise registration and global registration. Pairwise registration refers to the registration between two point-clouds collected by adjacent perspectives, and there are currently many methods [3,4,5] for this purpose. Global registration refers to the optimization of the registration accuracy from a global perspective based on the pairwise registration by eliminating the cumulative registration error. Global registration is the key technology for multi-view point cloud registration, and the registration result directly affects the performance of object reconstruction. However, the point cloud obtained by flash laser three-dimensional imaging lacks texture information and has severe outliers and noise, which cause great difficulties for global registration.

Global registration is a hot issue in the realm of point cloud processing, and a lot of research has been done: Steven et al. [6] proposed a global registration method for multi-density point clouds, which defined a kernel-based energy function that took all point clouds into account and distributed the errors evenly over the pairwise registration by estimating the surface kernel density. Simone et al. [7] globalized the Levenberg-Marquardt ICP (iterative closest point) method, unified the global registration error to an objective function, and then used the Jacobian matrix to derive the optimal solution. Kang et al. [8] used the corresponding points to realize global registration, which was robust to resolution differences. Zhu et al. [9] designed an evaluation function as the reconstruction accuracy criterion, roughly reconstructed an initial model, and then registered each point cloud to the initial model in a sequence. As a result, the global registration error was reduced by multiple iterations. Zhou et al. [10] established an objective function to minimize the distance between each corresponding point in the global point cloud, and they centralized the adjacent point cloud registration and global registration optimization in one step, which had high computational efficiency. Dorit et al. [11] proposed a graph optimization method to minimize global registration error, which has been widely used in simultaneous localization and mapping, but it required calculating the corresponding points between point clouds. Jochen et al. [12] proposed the explicit loop-closing technique, which separated the last scan of the closed-loop path from the previous scan and reaggregated it with explicit registration, so that the errors were evenly distributed throughout the registration process. However, it only had six degrees of freedom. Christian et al. [13] used the similarities in the entropy of adjacent registrations to achieve loop-closure detection, and then used graph optimization to achieve global registration. Based on Kinect Fusion [14], Li et al. [15] proposed a loop-closure detection method based on a historical model set, which had good real-time accuracy. Liu et al. [16] implemented loop-closure detection based on visual word bags, and they applied it to measuring the relative attitude of non-cooperative space targets. Liu et al. [17] did not need to specify loop-closure detection, but instead used two-way parametric registration to generate reversible transformations for global registration. This method was robust to pairwise registration errors, but sensitive to the outliers of pairwise registration. Arrigoni et al. [18] constructed block matrices and implemented global registration with LRS (low-rank sparse decomposition), but it was assumed that the submatrices were column-orthogonal to facilitate solving, which compressed the solution space and affected the accuracy and robustness, so this method was greatly affected by the sparsity of the registration matrix.

In general, the main goal of global registration is to construct the objective function and evaluation criteria for connecting corresponding points or determining the relationship between points and clouds, combined with closed-loop detection to establish constraints, and then to minimize the global registration error. Influenced by factors, such as ranging accuracy, backscattering, and distance change, the point clouds obtained by three-dimensional imaging using flash Lidar have severe noise and outliers, and undersampling often occurs while the distance between the Lidar and object is too large since the fix resolution of flash Lidar. These factors, especially outliers and undersampling, raise difficulties in searching for corresponding points between multi-view point clouds. Therefore, it is more suitable to use the transformation relationship between point clouds to achieve global registration. The theoretical basis of the commonly used global registration objective function is mainly divided into two types: Graph optimization and LRS. Graph optimization uses the relationship between the corresponding points in the multi-view point clouds and averages the error of each pairwise registration from the global perspective. LRS does not need to consider the corresponding points, and only relies on the pairwise registration relationship to separate the true pairwise registration from the pairwise registration with errors. LRS is more suitable for global registration of multi-view laser point clouds. However, LRS is rarely used in laser point cloud global registration, and there is still room for improvement of loop-closure detection and robustness to the noise of laser three-dimensional imaging.

In this study, loop-closure detection was realized according to the spatial distribution characteristics of the point cloud. Then, the corresponding weight matrix and transformation relation matrix of the multi-view point cloud were established, and the global registration objective function was constructed. The optimal solution of global registration was obtained by LRS, and orthogonalization projection of the optimal solution, as well as the registration relationships between the point clouds, were obtained. The following sections are arranged as follows: The second section explains the proposed method in detail. In the third section, the design of the corresponding simulated analyses is presented, the experiments are described, and the results are analyzed. In the fourth section, the algorithm is summarized. In the fourth section, the proposed method is summarized and prospects for areas that can be improved are discussed.

## 2. Study Method

**Figure 1:**
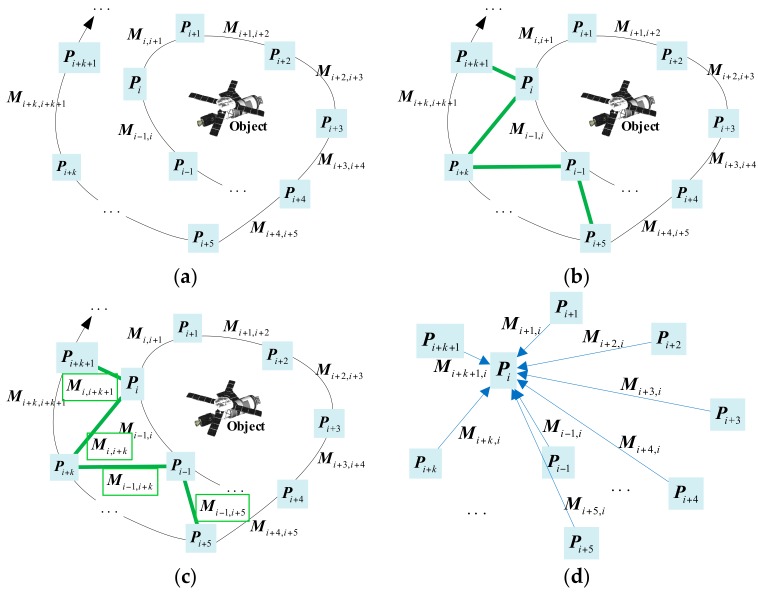
Global registration for single-object multi-view laser point cloud: (**a**) Pre-processing, pairwise registration between the consecutive point clouds; (**b**) loop-closure detection, searching the point clouds which may have several sets of transformation parameters; (**c**) post-preprocessing, pairwise registration between the new pairwise point clouds; (**d**) relation calculation, getting the transformation relationship between all point clouds and transforming them to the same coordinate system.

In moving object multi-view point cloud global registration, pairwise registration relationships between several point clouds are known, and the coordinates of any point cloud, $P_{i}$, (where i∈[1,n], n is the total number of point clouds) are considered the reference coordinate system. Then, the transformation relationship of other arbitrary point clouds, $P_{j}$, (where j∈[1,n]) to the reference coordinates must be found, as shown in Figure 1. Global registration can be divided into four steps: Pre-processing, loop-closure detection, post-preprocessing, and relation calculation. The green line in Figure 1b means the corresponding loop-closure point clouds founded in loop-closure detection.

The assumption of global registration is that any $P_{j}$ can obtain a transformation matrix between $P_{i}$ and $P_{j}$ directly or indirectly; that is, there are no isolated point clouds in the multi-view point cloud. As shown in Figure 1, assuming that the change relationship of $P_{i}$ to $P_{j}$ is $M_{ij}$, since there is a difference in the viewing angle between the cloud points of each viewpoint, the area of overlap is affected. In most cases, $P_{i}$ cannot be directly registered with any other $P_{j}$; that is, only some $M_{ij}$ values are known.

The unknown transformation relationship can also be obtained by continuous transformation of the intermediate point clouds from $P_{i}$ to $P_{j}$, but $M_{ij}$ generally has outliers and errors. Outliers can be rejected by use of robust estimators, but the noise will become more severe as the number of point clouds increases; a simple continuous transformation accumulates these outliers and errors. Global registration is a solution to the accumulation of outliers and errors. With these known transformation relations, combined with loop-closure detection, a global transformation relation matrix of point clouds is constructed, and the transformation relationship between each point cloud is considered globally. LRS decomposition of the transformation relation matrix obtains the estimated relationship, ${\hat{M}}_{ij}$, which minimizes the global registration total error. Equation (1) shows the generalized global registration objective function:

(1) $$f({\hat{M}}_{ij})=\min\sum_{i,j\in [1,n]}\left\|{\hat{M}}_{ij}-M_{ij}\right\|$$

### 2.1. Point Cloud Spatial Distribution Feature Extraction and Loop-Closure Detection

Loop-closure detection refers to finding point clouds that can be registered with more than one other dataset, yielding in different transformation parameters. Then, a new transformation parameter can be obtained with the pairwise registration of the point cloud obtained by loop-closure detection. The new transformation parameter forms a new constraint, which can effectively eliminate the error caused cumulatively by the continuous pairwise registration. Therefore, loop-closure detection is a necessary step in global registration. Moving object multi-view point clouds are in relative motion, and positions change between the system and the object at the time of acquisition. The point cloud of each viewpoint has severe laser noise, and there is a density difference between point clouds. It is necessary to evaluate the similarity of each point cloud.

**Figure 2:**
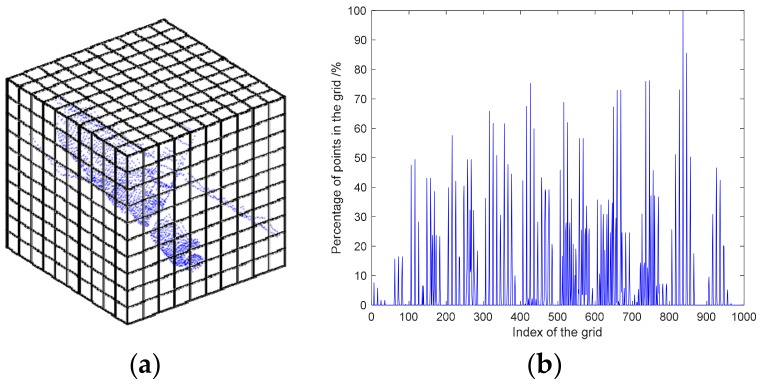
Schematic diagram of point cloud spatial distribution feature extraction: (**a**) Spatial rasterization, and (**b**) spatial distribution features of one point-cloud.

In this work, we constructed the spatial distributions for each point cloud, and the relationship between each point cloud was extracted by spatial distribution features to achieve closed-loop detection. As shown in Figure 2, the first point cloud, $P_{1}$, in the multi-view point cloud was used as a reference, and all the other point clouds were transformed by the known transformation, $M_{ij}$, to the coordinate system of $P_{1}$. The minimum bounding box, $B_{mmb\_all}$, which surrounded all point clouds, was extracted and rasterized into $n_{b}\times n_{b}\times n_{b}$ grids, and the grids were numbered from 1 to $n_{b}\times n_{b}\times n_{b}$. The quantity of points where $P_{i}$ falls in each grid was normalized, and the distribution histogram, $h_{hist}(i)$, of $P_{i}$ in space was obtained, where $h_{hist}(i)$ describes the spatial distribution characteristics of $P_{i}$. The distribution features of each point cloud in the grid can be regarded as the probability distribution characteristics of the point cloud in space. The points with similar spatial distribution features can be considered acquired when the viewpoint is similar, and the distribution feature relations between the point clouds are obtained. By correlating the distribution features, $h_{hist}(i)$, between the point clouds and removing the point cloud adjacent to $P_{i}$, the point cloud with distribution features similar to $P_{i}$ is the point cloud collected again from a viewpoint similar to $P_{i}$, and loop-closure detection is realized accordingly.

The point cloud distribution features obtained after spatial rasterization are not sensitive to point cloud density changes, and they showed robust performance when there are outliers and noise. The point clouds obtained by loop-closure detection were acquired from similar viewpoints; they have a high overlap rate and can be pairwise-registered to form a new constraint. For the point clouds of similar viewpoints, the similarity can be evaluated by their distribution features in space. The higher the similarity of distribution characteristics, the greater the similarity of the viewpoint, and the more accurate the pairwise registration. Equation (2) defines the similarity, $\Theta_{ij}$, between $P_{i}$ and $P_{j}$:

(2) $$\Theta_{ij}=\frac{\max(norm^{-1}[hist(i)-hist(j)])}{norm[hist(i)-hist(j)]}$$

### 2.2. Transformation Relation Matrix and Weight Matrix

Assuming $P_{i+k}$ is a point cloud from a viewpoint similar to $P_{i}$ obtained in loop-closure detection, then the transformation, $M_{i,i+k}$, from $P_{i}$ to $P_{i+k}$ forms a new constraint of the global registration. $M_{i,i-j}\ldots M_{i,i+j}$ and $M_{i,i+k-j}\ldots M_{i,i+k+j}$ can be obtained by pairwise registration for $P_{i}$, in which j is the maximum quantity of adjacent point clouds that can be registered. The value of j is determined by the adjacent point cloud overlap rate and the pairwise registration method. The higher the overlapping rate of adjacent point clouds, and the more robust the adjacent registration method, the larger the value of j.

According to the known transformation relationship, $M_{ij}$, between point clouds, the transformation relation matrix, $T_{global}$, is constructed as shown in Equation (3):

(3) $$T_{global}=\left(\begin{array}{cccc} I_{4} & M_{12} & \ldots & M_{12}\\ M_{12} & I_{4} & \ldots & M_{12}\\ \ldots & \ldots & \ldots & \ldots \\ M_{12} & M_{12} & \ldots & I_{4} \end{array}\right)$$

When $M_{ij}$ is unknown, $M_{ij}$ in $T_{global}$ is an all-zero matrix. The more constraints obtained by closed-loop detection, the denser the matrix, $T_{global}$, and the better the performance of the global registration. Considering the existence of outliers in pairwise registration, combined with the known condition that $M_{ij}$ and $M_{ji}$ are mutually reversible matrices, $M_{ij}$ and $M_{ji}^{-1}$ are considered to be correct only when the difference between $M_{ij}$ and $M_{ji}$ is below a threshold. Otherwise, they are considered to be inaccurate registrations, and all values in $M_{ij}$ and $M_{ji}$ are set to zero.

The weight matrix, $W_{weight}$, is the same size as $T_{global}$, and the purpose of constructing $W_{weight}$ is to make the objective function only work on valid values in $T_{global}$, which can reduce the effect of the sparsity caused by element deletion in $T_{global}$ on global registration. Equation (4) shows the value of element $w_{ij}$ in $W_{weight}$:

(4) $$w_{ij}=\{\begin{array}{ll} \Theta_{ij} & ,t_{ij}\neq 0\\ 0 & ,t_{ij}=0 \end{array}$$

### 2.3. Objective Function Construction

**Figure 3:**
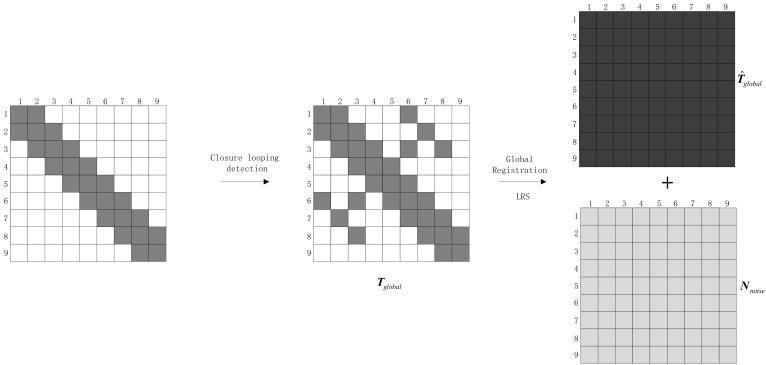
Schematic diagram of global registration based on LRS decomposition.

Outliers and errors are inevitable in pairwise registrations, and the transformation relationships in $T_{global}$ are not always accurate. $T_{global}$ can be decomposed into the following equation:

(5) $$T_{global}={\hat{T}}_{global}+N_{noise}$$

where ${\hat{T}}_{global}$ is the estimated value matrix of the accurate registration, and it is also the expected target in global registration; $N_{noise}$ is the noise matrix, i.e., the outliers and errors of registration existing in $T_{global}$. The element in $T_{global}$ is only partially known, and $T_{global}$ is a sparse matrix; the block element, $M_{ij}$, in $T_{global}$ is a rigid body transformation with a rank of 4, thus, the rank of $T_{global}$ is also 4, so $T_{global}$ is a sparse low-rank matrix. The purpose of global registration is to complement the missing elements in $T_{global}$ under the constraints of known noisy observations. From the perspective of matrix analysis—that is, when the rank of the target matrix is known—the unknown matrix information is complemented by the known rank constraint and the limited known matrix elements. This is a low-rank sparse matrix completion problem, as shown in Figure 3.

The pairwise registration of adjacent point clouds in the preprocessing usually does not cause errors because their overlap is enough; but it cannot guarantee that the point cloud obtained by closed loop detection are all completely correct, as there may be false results in the loop-closure detections and the pairwise registration between them may be wrong, resulting in the outliers in $T_{global}$. Considering that the $L_{1}$ norm is more robust than $L_{2}$ norm when there are missing values and outliers, we used the $L_{1}$ norm to evaluate the difference between $T_{global}$ and ${\hat{T}}_{global}$. To solve this, ${\hat{T}}_{global}$ is usually bilinearly decomposed into two matrices, U and V. Equation (6) shows the objective function and constraints.

(6) $$\{\begin{array}{l} \underset{{\hat{T}}_{global}}{\arg\min}{\left\|W_{weight}⊙(T_{global}-{\hat{T}}_{global})\right\|}_{1}\\ {\hat{T}}_{global}=UV^{T}\\ rank({\hat{T}}_{global})=4 \end{array}$$

where $rank({\hat{T}}_{global})$ is the rank of ${\hat{T}}_{global}$.

However, the minimization of Equation (6) is a non-convex discontinuous problem. It is necessary to introduce a relaxation term to normalize it, and the introduction of the relaxation term cannot have a great impact on the value of the original objective function. We used the matrix completion method in [19] to normalize the objective function, as shown in Equation (7).

(7) $$\underset{U,V}{\arg\min}[{\left\|W_{weight}⊙(T_{global}-UV)\right\|}_{1}+\frac{\lambda }{2}({\left\|U\right\|}_{F}^{2}+{\left\|V\right\|}_{F}^{2})]$$

where λ is the weight parameter of the relaxation term, which is used to control the relationship between the observed value and the estimated value. When λ is small, the obtained estimated value, ${\hat{T}}_{global}$, describes the observed value more accurately, but its prediction effect is not good and it over-optimizes; when λ is large, the obtained estimated value, ${\hat{T}}_{global}$, is inaccurately described for the observed value, and it is under-optimized. The empirical value of λ is generally $\lambda \in [1e^{-1},1e^{-7}]$. The constructed objective function has no orthogonality constraints on U and V, which exceeds the limit of six degrees of freedom, so it does not compress the solution space. Compared with existing methods, the proposed method is more in line with the actual situation of single-object multi-view global registration, and it has the advantage of robustness.

### 2.4. LRS Decomposition by Augmented Lagrange Method

Equation (7) is equivalent to Equation (8), which we solved using the Augmented Lagrange method.

(8) $$\underset{{\hat{T}}_{global},U,V}{\arg\min}{\left\|W_{weight}⊙(T_{global}-{\hat{T}}_{global})\right\|}_{1}+\frac{\lambda }{2}({\left\|U\right\|}_{F}^{2}+{\left\|V\right\|}_{F}^{2})$$

The Augmented Lagrange method adds a penalty term based on the Lagrange method to obtain the solution. The corresponding Augmented Lagrange function of Equation (8) is Equation (9).

(9) $$\begin{array}{c} f({\hat{T}}_{global},U,V)={\left\|W_{weight}⊙(T_{global}-{\hat{T}}_{global})\right\|}_{1}+\frac{\lambda }{2}({\left\|U\right\|}_{F}^{2}+{\left\|V\right\|}_{F}^{2})\\ +\langle L,{\hat{T}}_{global}-UV\rangle +\frac{\mu }{2}{\left\|{\hat{T}}_{global}-UV\right\|}_{F}^{2} \end{array}$$

where L is a Lagrange multiplier matrix for the iterative solution; μ is a penalty factor; and 〈A,B〉 is defined as a trace of $A^{T}B$. The minimization of $f({\hat{T}}_{global},U,V)$ generally uses the Gauss–Seidel method to iteratively solve ${\hat{T}}_{global}$, U, and V. The solution of Equation (9) is decomposed into three minimization subproblems; that is, each iteration is divided into three steps that are mutually constrained. For example, the mth iteration is as follows:

(0) Parameter:

(10) $$\left\{ \begin{array}{l} \mu_{m}=\rho \mu_{m-1}\\ L_{m}=L_{m-1}+\mu_{m-1}({\hat{T}}_{global}-UV) \end{array}\right.$$

where ρ is a constant parameter used to adjust the convergence speed. The larger the value of ρ, the closer the value of ${\hat{T}}_{global}$ to UV, which speeds up the convergence of the algorithm, but it simultaneously affects the accuracy of the algorithm to a certain extent. In this paper, ρ=1.05 and $\mu_{0}=1\times 10^{-6}$, and all elements in $L_{0}$ are $1\times 10^{-12}$.

(1) With fixed ${\hat{T}}_{global}$ and V, solve U.

Then, Equation (9) can be expressed as a function of U, as shown in Equation (11).

(11) $$f(U)\propto \frac{\lambda }{2}{\left\|U\right\|}_{F}^{2}+\langle L,{\hat{T}}_{global}-UV\rangle +\frac{\mu }{2}{\left\|{\hat{T}}_{global}-UV\right\|}_{F}^{2}$$

When the derivative of Equation (11) on U is 0, ζ(U) takes the minimum value, and U is as shown in Equation (12).

(12) $$U=(\mu {\hat{T}}_{global}+L)V(\mu V^{T}V+\lambda I_{4})$$

(2) With fixed ${\hat{T}}_{global}$ and U, solve V.

Next, Equation (9) can be expressed as a function of V, as shown in Equation (13).

(13) $$f(V)\propto \frac{\lambda }{2}{\left\|V\right\|}_{F}^{2}+\langle L,{\hat{T}}_{global}-UV\rangle +\frac{\mu }{2}{\left\|{\hat{T}}_{global}-UV\right\|}_{F}^{2}$$

When the derivative of Equation (11) on V is 0, ζ(U) takes the minimum value, and V is as shown in Equation (14).

(14) $$V={\left(\mu {\hat{T}}_{global}+L\right)}^{T}U{\left(\mu U^{T}U+\lambda I_{4}\right)}^{-1}$$

(3) With fixed U and V, solve ${\hat{T}}_{global}$.

Then, Equation (9) can be made equivalent to Equation (15).

(15) $$\underset{Z}{\min}{\left\|W_{weight}⊙(T_{global}-{\hat{T}}_{global})\right\|}_{1}+\frac{\mu }{2}{\left\|{\hat{T}}_{global}-UV+\mu^{-1}L\right\|}_{F}^{2}$$

Equation (15) can be solved by an element-by-element shrink operation [20]. Then, Equation (16) shows the equation for ${\hat{T}}_{global}$.

(16) $${\hat{T}}_{global}=W_{weight}⊙(T_{global}-S_{\mu^{-1}}(T_{global}-UV-\mu^{-1}L))+{\bar{W}}_{weight}⊙(UV-\mu^{-1}L)$$

where ${\bar{W}}_{weight}$ is the complement of $W_{weight}$. The contraction operator, $S_{\varepsilon }(x)$, is as shown in Equation (17).

(17) $$S_{\varepsilon }(x)=\{\begin{array}{ll} x-\varepsilon & ,x>\varepsilon \\ x+\varepsilon & ,x<-\varepsilon \\ 0 & ,-\varepsilon \leq x\leq \varepsilon \end{array}$$

When the difference between ${\hat{T}}_{global}$ and UV is sufficiently small, the iteration is terminated, and the registration relationship matrix, ${\hat{T}}_{global}$, can be obtained. Considering that the diagonal block matrix, $M_{ii}$, of the matrix, UV, is a transformation relationship between the point cloud and itself, in the ideal case, all of the $M_{ii}$ values should be equivalent to $I_{4}$. The final iteration termination condition is as shown in Equation (18).

(18) $$\{\begin{array}{l} {{\left\|{\hat{T}}_{global}-UV\right\|}_{F}/\left\|W_{weight}⊙T_{global}\right\|}_{F}\leq e_{threshold\_iteration}\\ \left|\mathrm{tr}(UV-I_{4n})\right|\leq 4n\cdot e_{threshold\_optimal} \end{array}$$

where $\mathrm{tr}(UV-I_{4n})$ is the trace of $UV-I_{4n}$, and $e_{threshold\_iteration}$ and $e_{threshold\_optimal}$ are determined by the accuracy of the pairwise registration. In this paper, $e_{threshold\_iteration}=1\times 10^{-9}$ and $e_{threshold\_optimal}=1\times 10^{-8}$, which can serve as a reference.

### 2.5. Block Matrix Orthogonalization

In the ideal case, where there are no influences, such as outliers and errors, each of the 4 × 4 block matrices, ${\hat{M}}_{ij}$, in the matrix, ${\hat{T}}_{global}$, obtained in Section 2.4 is a registration matrix of $P_{i}$ to $P_{i+j}$. However, considering the noise caused by outliers and errors, ${\hat{M}}_{ij}$ obtained by low-rank sparse decomposition generally does not satisfy the orthogonal constraints required for rigid body transformation, and it needs to be projected into orthogonal space. 

The first step is to normalize ${\hat{M}}_{ij}$, assuming that the element in ${\hat{M}}_{ij}$ is ${\hat{m}}_{ij}$. Equation (19) shows the normalization of ${\hat{M}}_{ij}$.

(19) $${\hat{M}}_{ij}={\hat{M}}_{ij}/{\hat{m}}_{4,4}$$

Then, the 0th, 1st, and 3rd row elements of the 4th column are set to 0 to satisfy the constraint of the rigid body transformation, as shown in Equation (20).

(20) $${\hat{m}}_{ij}=\{\begin{array}{llll} 0 & ,1\leq i\leq 3 & and & j=4\\ {\hat{m}}_{ij} & ,i=4\\ {\hat{r}}_{ij} & ,1\leq i\leq 3 & and & 1\leq j\leq 3 \end{array}$$

where ${\hat{r}}_{ij}$ is an element in the 3 × 3 matrix, ${\hat{R}}_{ij}$, which is defined in Equation (21).

(21) $${\hat{R}}_{ij}=U_{{\hat{M}}_{33}}QV_{{\hat{M}}_{33}}^{T}$$

where Q is a 3 × 3 diagonal matrix, and the diagonal elements are 1, 1, and $\left|U_{{\hat{M}}_{33}}V_{{\hat{M}}_{33}}^{T}\right|$, respectively. Equation (22) shows the definition of $U_{{\hat{M}}_{33}}$ and $V_{{\hat{M}}_{33}}$.

(22) $$[U_{{\hat{M}}_{33}},S_{{\hat{M}}_{33}},V_{{\hat{M}}_{33}}]=svd\left[\begin{array}{ccc} {\hat{m}}_{11} & {\hat{m}}_{12} & {\hat{m}}_{13}\\ {\hat{m}}_{21} & {\hat{m}}_{22} & {\hat{m}}_{23}\\ {\hat{m}}_{31} & {\hat{m}}_{32} & {\hat{m}}_{33} \end{array}\right]$$

The obtained 4 × 4 block matrix, ${\hat{M}}_{ij}$, in ${\hat{T}}_{global}$ is the registration matrix of $P_{i}$ to $P_{j}$. The registration matrix of any $P_{i}$ to $P_{j}$ under the global optimization condition can be obtained, and the global registration is completed.

## 3. Experiments and Analysis

**Figure 4:**
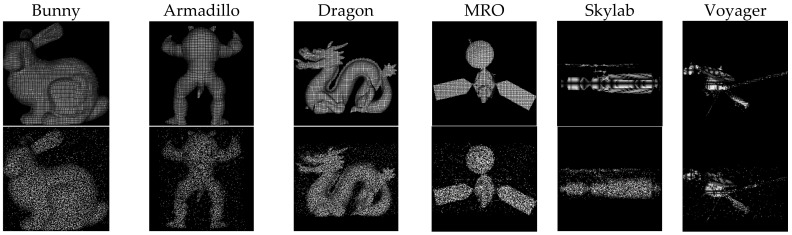
The point clouds with added Gaussian noise and outliers: The point clouds in the first row are the original point clouds, the second row are the first row with added outliers and noise.

The experiments were divided into a simulation point cloud analysis and a real point cloud test. In Section 3.1 and Section 3.2, the simulation point clouds were used to control the variables to analyze the performance of the proposed method. In Section 3.3, the real point clouds were used to comprehensively test the proposed method’s practical performance. The simulation point clouds were simulated by an array plane 3D camera based on the time-of-flight (TOF), as described in [21]. During the process of generating a simulated point cloud with continuous adjacent angles of view, there was a relative position transformation between the object and the system to simulate the motion of the object. The ratio of the maximum and minimum distance between the system and the object in adjacent point clouds was 2:1, and the angle differences between adjacent point clouds was fixed at 20°. Outliers and Gaussian noise were added to simulate laser three-dimensional imaging noise. The quantity of outliers was 0.25 times the quantity of points in each point cloud; the mean of the Gaussian noise was 0, and the variance was 0.01 times the length of the minimum bounding box diagonal. Noise and outliers were added primarily to analyze the robustness of the loop-closure detection, and we assumed that the pairwise registrations between continuous point clouds in the preprocessing were achievable. Six models were selected as objects: Bunny [22], Armadillo [23], and Dragon [24] from Stanford; MRO (Mars reconnaissance orbiter), Skylab, and Voyager from NASA (National Aeronautics and Space Administration) [25]. Bunny, Armadillo, and Dragon had rich structural information, while space targets, MRO, Skylab, and Voyager, had relatively simple structural information, such as numerous repetitions and similar structures. Figure 4 shows the rendered point clouds based on shading; the first row is the front view of the point clouds of the six objects, and the second row was the corresponding point cloud after adding the outliers and Gaussian noise to the point cloud of the first line, which is also the simulation point cloud used in our simulation analysis.

### 3.1. Loop-Closure Detection Accuracy Analysis

The point cloud distribution feature histogram is related to the quantity of grids. At the same time, the pairwise registration error of the point cloud also affects the point cloud distribution feature, which directly affects the loop-closure detection accuracy. To test the robustness of the proposed loop-closure detection method, the correlation between the loop-closure detection accuracy, the quantity of grids, and the error of pairwise registration was analyzed.

**Figure 5:**
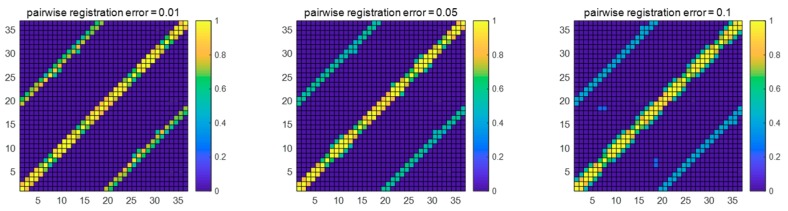
The weight matrixes under three different pairwise registration errors

Figure 5 shows the weight matrix under three different adjacent registration errors. In the simulation, the object rotated 20° continuously around the fixed axis. Therefore, under ideal conditions, the point cloud of the ith view should form a closed loop with the point cloud of the i±17th view. Figure 5 shows that when the pairwise registration error was 0.01 rad, the cumulative error was relatively small. The proposed method could complete loop-closure detection, and the similarity of the point cloud obtained by loop-closure detection was relatively high, so the weight of the transformation relationship was also relatively high. When the pairwise registration errors were 0.05 rad and 0.1 rad, the cumulative error became severe as the pairwise registration error increased, and the point cloud weights obtained by loop-closure detection decreased. There were four detection errors at 0.1 rad.

**Figure 6:**
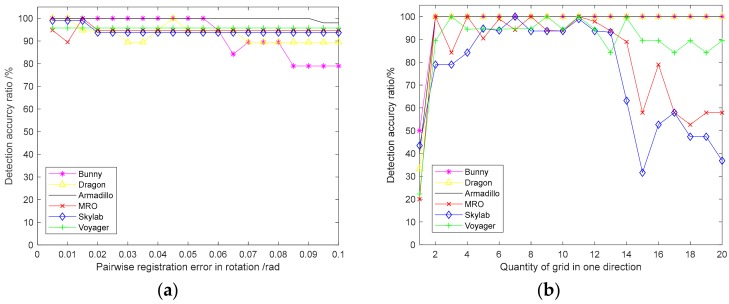
Relationship between detection accuracy and related factors. (**a**) The relation between detection accuracy and the pairwise registration error; (**b**) the relation between detection accuracy and the quantity of grids.

Figure 6a shows the relationship between the loop-closure detection accuracy and the pairwise registration error. When the pairwise registration error was below 0.055 rad, the loop-closure detection accuracy of the six point-clouds was over 90%. However, as the registration error increased further, the accuracy began to decline. Because of the repetition or similar structures of the three space targets, the accuracy was more susceptible to the adjacent registration error. This shows that the proposed loop-closure detection method has certain requirements for the registration error of adjacent frames, one of them being that it should not be too large.

Figure 6b shows the relationship between the quantity of grids and the loop-closure detection accuracy. When the quantity of grids was too small, the loop-closure detection accuracy was correspondingly low because it could not express the detailed distribution feature of each point cloud. When the quantity of grids was too large, the loop-closure detection accuracy also decreased because it expressed the point cloud distribution feature with too much detail. When the quantity of grids was between $5^{3}$ and $12^{3}$, the loop-closure detection accuracy of the six point-clouds reached more than 90%, which indicates that the proposed loop-closure detection method can achieve good results within a wide range of the quantity of grids. Figure 6 also shows that MRO, Skylab, and Voyager were more sensitive to changes in the quantity of grids, which is due to the fact that there were more repeats or similar structures in the three space targets. When the quantity of grids was not appropriate, the impact was more obvious.

### 3.2. Simulation Point Cloud Analysis

With the same simulation point cloud as in Section 3.1, the proposed method was used in its entirety to analyze the relationship between global registration, pairwise registration outliers, and errors. The incremental registration, the LUM [11] in the PCL (Point Cloud Library) library [26], and the LRS-based RegL1 algorithm in [18] were selected as the methods to compare with the proposed method. Considering that the loop-closure detection method was not given in [13], the proposed loop-closure detection method described in this paper was used with the RegL1 method, but the assignment of elements in the weight matrix still adopted the method in [18]. Additionally, both of the four kinds of global registration method were based on the same pairwise registration method and the same termination in the consecutive point clouds pairwise registration to minimize the influence of the pairwise registration result.

#### 3.2.1. Global Registration Accuracy of a Single Object

**Figure 7:**
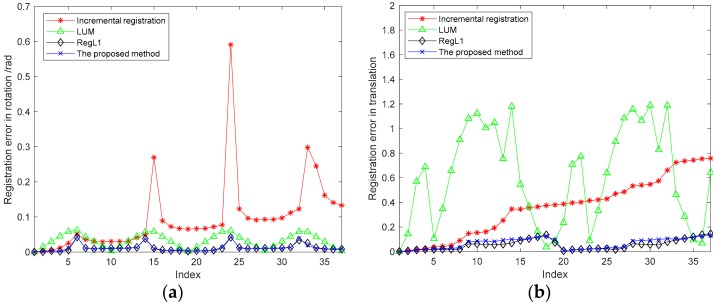
The relation between registration error and the index of view. (**a**) Registration error in rotation; (**b**) registration error in translation.

When the pairwise registration error was 0.01 rad, 37 consecutive point clouds of a single object were globally registered by the four different methods, and Figure 7 shows the global registration errors of the respective viewpoints. Figure 7 also shows that, with the increase of the point cloud index, the incremental registration method formed an obvious cumulative registration error, and LUM averaged the cumulative error to a certain extent. RegL1 and the proposed method had better performances.

#### 3.2.2. Relationship between Global Registration and Pairwise Registration Outliers

**Figure 8:**
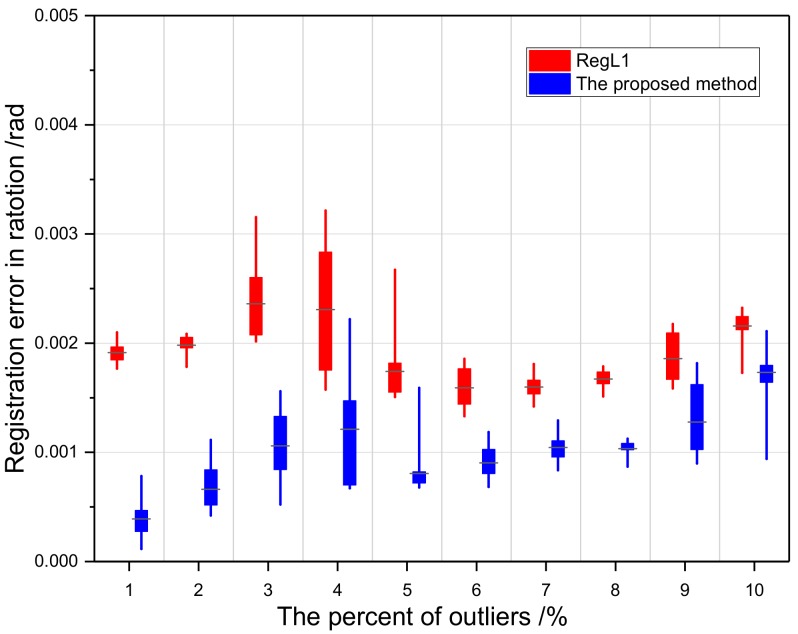
The relation between registration error and the percentage of outliers.

When there were errors during loop-closure detection, the detected false neighboring point cloud overlap rate was low. A low overlap rate might lead to a wrong pairwise registration, which might result in an outlier in $T_{global}$. The proposed loop-closure detection method had an accuracy of over 90% under most parameter conditions. Therefore, the performance of the proposed method with 1–10% outliers was tested. We replaced the correct transformation matrix, $M_{ij}$, of the random position with the corresponding proportion of a 4 × 4 unit matrix, and Figure 8 shows the experimental results. When the percentage of outliers was 1–10%, the performance of the proposed method was relatively robust, the global registration error was lower than 0.005 rad, and the overall performance was better than RegL1.

#### 3.2.3. Relationship between Global Registration and Pairwise Registration Errors

**Figure 9:**
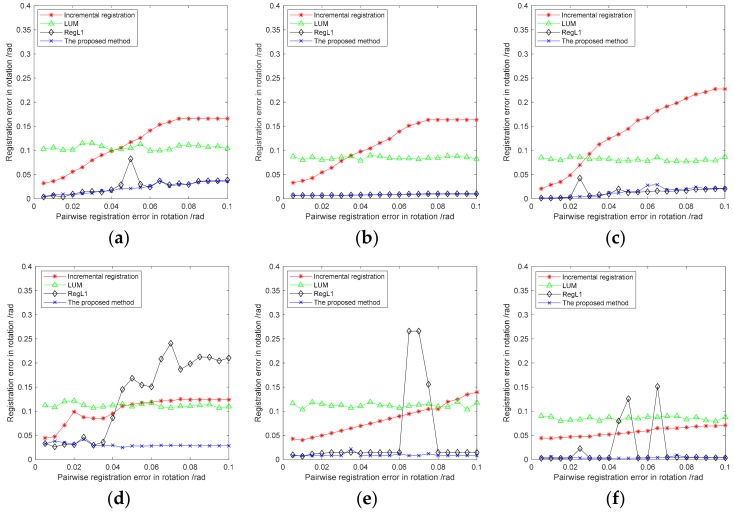
The relation between registration error and the pairwise registration error: (**a**) Bunny, (**b**) Armadillo, (**c**) Dragon, (**d**) MRO, (**e**) Skylab, and (**f**) Voyager.

The results of global registration were also affected by pairwise registration errors. Figure 9 shows the performance of the four methods with different pairwise registration errors. The global registration error represented by the curve in Figure 9 was the average of the registration errors of all point clouds after global registration with the current pairwise registration error. Figure 9 also shows that the incremental registration method tended to increase the error as the pairwise registration error increased. When the pairwise registration error reached a certain level, the incremental registration error tended to be stable. LUM averaged the error caused by pairwise registration to a certain extent, but the overall performance was not optimal. RegL1 and the proposed method had better performances on Bunny, Armadillo, and Dragon, but the performance of the proposed method was more robust. RegL1 and the proposed method had some serious global registration errors with MRO, Skylab, and Voyager. This is because when the pairwise registration error increased, the probability of the error during loop-closure detection increased, which led to more outliers and errors in $T_{global}$ at the same time. The three space-point clouds were more severely affected due to their simple structures, which resulted in a more rigorous test for the stability of the global registration method. However, RegL1 had a larger number of severe global registration errors than the proposed method in general, which we presume to be caused by the compression of the solution space by RegL1. The proposed method was more robust due to the more reasonable objective function and the corresponding weight matrix. When the pairwise registration error was 0.01–0.1 rad, the global registration error of the proposed method was less than 0.05 rad in most cases.

Table 1 shows the global registration results of each method applied to six kinds of point clouds when the pairwise registration error was 0.03 rad. We chose the point clouds at 0° and 360° because, in the ideal condition, they were completely overlapping, and the Euclidean distance between the corresponding points should be equal to 0. We filtered out the outliers in the point clouds, calculated the distance between the corresponding points after registration, and tinted them according to the distance value. The corresponding residuals are below the results. The four global registration methods could optimize the pairwise registration results to a certain extent, but in the case of severe noise and outliers in these point clouds, the LUM had difficulty in finding corresponding points. The LRS-based RegL1 and our method were relatively robust, and our method performs best overall.

### 3.3. Experimental Test and Result

The proposed method was further tested with a real point cloud. The point clouds were obtained by an array plane 3D imaging device based on the TOF principle. The models were diffuse reflection space models, such as the Apollo, Skylab, and Tian-gong models. The models were placed on a rotary table with a controllable rotation angle, the object was rotated after each acquisition, and the rotation angle was fixed at 20°. A total of 37 point-clouds were acquired, which is consistent with the simulation point cloud parameter settings. The point clouds with an odd index were twice the imaging distance of the adjacent point clouds with an even index. Thirty-seven point-clouds of an object were transformed in the same coordinate system, since the global registration relationship between real point clouds is not exactly clear, the point clouds after transformation were sliced at the same position. Table 2 shows the global registration results and the slice results of the four methods.

Table 2 shows that there were serious outliers and noise in the three real point clouds, and the four methods were affected to different degrees. There were certain differences between the global registration results of the real point clouds and the simulated point cloud. The reason for these differences is that the outliers and noise affected the results of the pairwise registration, which in turn affected the results of the global registration. In this case, Table 1 shows that LUM did not obtain the optimal solution for Apollo and Skylab, but the results for Tian-gong were good. RegL1 failed to register with Skylab, but the results for Apollo and Tian-gong were good. The proposed method obtained fine edges of the three kinds of point clouds, and the contour information of the object was clear, which shows the robustness of the proposed method when applied to a real point cloud.

## 4. Conclusions

In this paper, a multi-view laser point cloud global registration method based on low-rank sparse decomposition was proposed. The spatial distribution features of each point cloud were extracted by spatial rasterization, and loop-closure detection of multi-view point clouds was realized according to the similarity of spatial distribution features. According to the similarity of point clouds obtained by loop-closure detection, the corresponding weight matrix was designed, which enhanced the robustness of the global registration to sparseness and errors in the transformation relation matrix. The objective function that satisfied the global optimization condition was constructed. In the regularization process, the compression solution space problem caused by the column-orthogonal hypothesis of the matrix was avoided, which made the solution more realistic and more robust. The objective function was solved by the Augmented Lagrange method, and the iterative termination condition was designed by considering the iterative error and the a priori condition of global registration. The simulation analysis proved that the proposed loop-closure detection method was robust with a wide range of preset parameters, and the accuracy of loop-closure detection was over 90%. When there were outliers in the pairwise registration, the performance of the proposed method was better than that of RegL1. When the pairwise registration error was below 0.1 rad, the proposed method performed better than the three other methods, and the global registration accuracy was 0.05 rad. Finally, further experiments were carried out with real point clouds. The validity and robustness of the proposed method were further proved by the global registration results and slice results.

In general, the proposed method is robust with a wide range of preset parameters, and is less affected by density changes and noise. These advantages make it suitable for global registration of moving object multi-view laser point clouds, and it has a relatively high application value. However, there are some shortcomings in the proposed method. For example, for the three kinds of space target point clouds with repeated structures, the accuracy of the proposed method declined to some extent, which indicates that the loop-closure detection method has certain requirements for the object structure. At the same time, the proposed method has certain requirements for the error of pairwise registration. The proposed method can also be used by multiple iterations to further improve the global registration accuracy.

## Figures and Tables

**Table 1:** The registration result with different methods on simulated point clouds.

	Bunny	Armadillo	Dragon	MRO	Skylab	Voyager	
**Increment ICP**	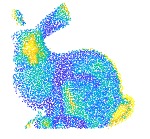	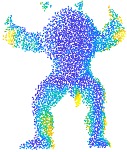	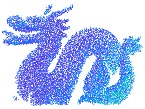	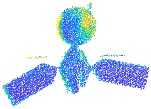	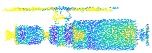	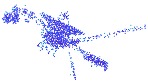	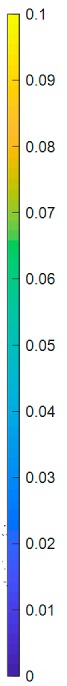
	0.0376	0.0288	0.0169	0.0250	0.0517	0.0136	
**LUM**	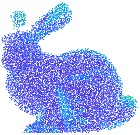	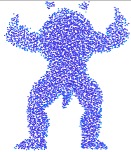	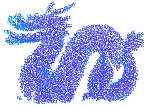	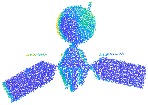	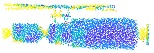	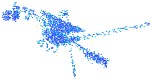	
	0.0139	0.0140	0.0126	0.0196	0.0368	0.0215	
**RegL1**	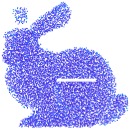	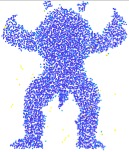	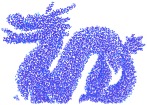	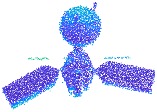	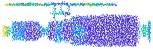	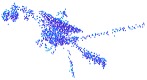	
	0.0101	0.0112	0.0108	0.0152	0.0170	0.0132	
**Ours**	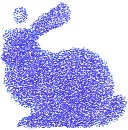	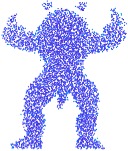	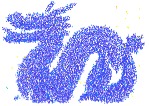	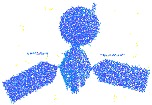	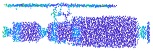	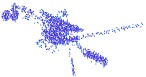	
	0.0098	0.0112	0.0108	0.0149	0.0128	0.0130	

**Table 2:** The registration result with different methods on real point clouds.

	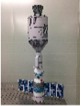 Apollo	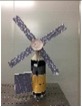 Skylab	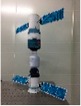 Tian-Gong
**Increment ICP**	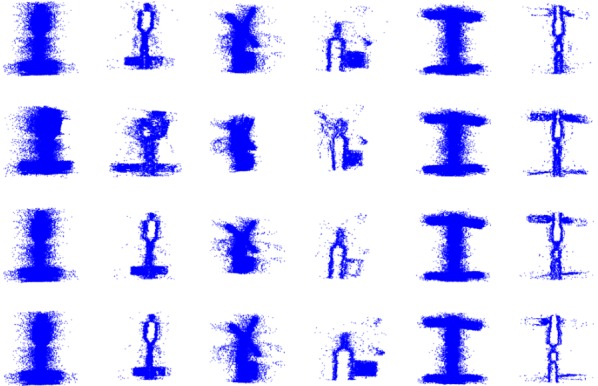		
**LUM**			
**RegL1**			
**The Proposed Method**			
